# Identification of genes and haplotypes that predict rheumatoid arthritis using random forests

**DOI:** 10.1186/1753-6561-3-s7-s68

**Published:** 2009-12-15

**Authors:** Rui Tang, Jason P Sinnwell, Jia Li, David N Rider, Mariza de Andrade, Joanna M Biernacka

**Affiliations:** 1 Department of Health Sciences Research, 200 First Street Southwest, Mayo Clinic, Rochester, Minnesota 55905, USA

## Abstract

Random forest (RF) analysis of genetic data does not require specification of the mode of inheritance, and provides measures of variable importance that incorporate interaction effects. In this paper we describe RF-based approaches for assessment of gene and haplotype importance, and apply these approaches to a subset of the North American Rheumatoid Arthritis Consortium case-control data provided by Genetic Analysis Workshop 16. The RF analyses of 37 genes identified many of the same genes as logistic regression, but also suggested importance of certain single-nucleotide polymorphism and genes that were not ranked highly by logistic regression. A new permutation method did not reveal strong evidence of gene-gene interaction effects in these data. Although RFs are a promising approach for genetic data analysis, extensions beyond simple single-nucleotide polymorphism analyses and modifications to improve computational feasibility are needed.

## Background

Although it is expected that gene-gene interactions contribute to complex traits, most studies assess genetic associations with one single-nucleotide polymorphism (SNP) or one gene at a time. Model-based methods for studying gene-gene interactions in genome-wide association studies have been proposed [1], but these methods exacerbate multiple-testing problems and are computationally challenging. Moreover, they are generally used to test only for two-way interactions that over-simplify the disease-causing mechanisms.

Random forests (RFs) have been proposed as an alternative strategy for the analysis of genetic data [2-5]. Although RFs are not well suited for assessment of statistical significance [6], they may prove useful for prioritizing SNPs or genes for further study. RFs can be applied when many potential predictors exist, and have good predictive performance. More importantly, these methods do not require specification of the mode of inheritance, and assessment of variable importance (VI) incorporates interaction effects. RFs are usually fitted using single SNPs as predictors, and single-SNP importance measures are calculated. Consideration of haplotype [7,8] or gene-level importance measures may reveal additional effects.

In this paper we describe novel uses of the RF approach for the assessment of gene and haplotype importance, and apply the proposed approaches to the detection of genes containing variations that predict rheumatoid arthritis (RA).

## Methods

### Data

We analyzed genotypes of 868 cases and 1194 controls from the North American Rheumatoid Arthritis Consortium (NARAC) Genetic Analysis Workshop 16 data for 17 candidate genes selected on the basis of a literature search (*PADI4*, *PTPN22*, *ITGAV*, *STAT4*, *IL1B*, *CTLA4*, *IL13*, *LTA*, *HLA-A*, *HLA-B*, *HLA-C*, *VEGFA*, *TNF*, *IL6*, *C5*, *TRAF1*, and *MS4A1*) as well as 20 "null" genes not suspected to have an impact on RA risk. SNPs with low call rates (<95%) or not in Hardy-Weinberg equilibrium (*p *< 0.001) were excluded. In addition, random subsets of SNPs were excluded from several large null genes, leading to 135 candidate-gene SNPs and 110 null-gene SNPs. For haplotype analyses tag SNPs were selected for each gene using H-clust http://wpicr.wpic.pitt.edu/WPICCompGen/hclust/hclust.htm. MACH [9] was used to impute missing genotypes before analysis.

### Basic RF analysis

The RF method implemented in the R package randomForest [10] was used to analyze 245 SNPs in 37 genes with RA case/control status as the outcome. For a detailed description of the RF approach, see Breiman [6]. We used the default tuning parameter *m*_*try *_= , where *p *is the total number of predictor variables (*p *= 245) and generated forests with 5000 trees. Our results are based on the scaled mean decrease in accuracy measure of VI. We first used SNP genotypes coded as 0, 1, or 2 as predictors and ranked SNPs based on the single-SNP VI. Subsequently, we considered novel ways of assessing gene and haplotype importance.

### Application of RFs for studying gene and haplotype effects

To assess gene importance based on SNP VI measures, we ranked genes according to 1) the maximum SNP VI over all SNPs in a gene, or 2) the mean SNP VI for a gene. Further, we implemented a RF analysis of haplotype effects, similar to that proposed by Nonyane and Foulkes [8]. The function Haplo.EM [11] in the statistical package R was used to estimate the posterior probabilities of all possible haplotype pairs in each gene, for each person, given their genotypes. A pair of haplotypes in each gene was then drawn for each person according to these probabilities, and a RF with 10 trees was grown. This process of randomly selecting haplotypes from their posterior distributions and fitting a RF of 10 trees was repeated 100 times. The 100 sets of 10 trees were then combined, and the VI of each haplotype was calculated using the full ensemble of 1000 trees. Measures of haplotype importance were used to calculate gene importance in a way similar to using SNP importance measures (i.e., based on the maximum or mean haplotype VI for a gene).

### A permutation approach for investigating gene-gene interactions

To assess the impact of genetic variations not only as predictors of RA but also as modifiers of the effects of other genes, we propose a gene-permutation approach. We first fit a RF to the original data and obtain a measure of VI of each SNP in each gene (*VI*_*g*, *i*_, *g *= 1,...*G*, *i *= 1,...,*n*_*g*_, where *G *is the number of genes and *n*_*g *_is the number of SNPs in gene *g*). We then permute the genotypes at all SNPs within a gene of interest among the subjects. Genotypes at all SNPs within a gene are permuted together, maintaining the LD structure within that gene. After permuting gene *k *we fit a RF to the resulting data, and recalculate the VI measures of all SNPs (, where *g *and *i *are defined as above and *k *denotes the permuted gene, *k *= 1,...,*G*). Finally, we calculate the difference of pre- and post-permutation SNP VI measures (, *k *= 1,...,*G*, *g *= 1,...,*G*, *i *= 1,...,*n*_*g*_). By permuting genotypes at all SNPs in a gene, the effect of the gene and all of its interactions are removed, leading to a decrease in the VI of SNPs in the permuted gene (if it is associated with the disease) as well as possibly any gene that interacts with the permuted gene. Therefore, for a SNP in the permuted gene (*k *= *g*), the difference in pre- and post-permutation VI assesses importance of the SNP, whereas for a SNP not in the permuted gene (*k *≠ *g*), a decrease in VI may reveal gene-gene interactions. Conversely, SNPs in genes in LD with the permuted gene are expected to increase in importance after permutation.

To demonstrate the utility of this permutation procedure, we applied it to two simulated datasets consisting of genotypes at ten unlinked SNPs for 100 cases and 100 controls. Both datasets were generated assuming two biallelic risk loci: SNP 1 with alleles a and A with *p*(A) = 0.60 and SNP 2 with alleles b and B with *p*(B) = 0.33. Genotypes were randomly generated from these allele frequencies assuming Hardy-Weinberg equilibrium. Affected status was then randomly generated conditional on the genotype assuming the following penetrance matrices:

The first 100 cases and 100 controls were retained for analysis. For the dataset generated under Model 1, allele A at SNP1 had a relative risk of 1.5 regardless of the genotype at SNP2, and allele B at SNP2 had a relative risk of 2 regardless of the genotype at SNP1. Model 2 represents a situation with weak marginal effects of SNPs 1 and 2, and a strong interaction between, the two SNPs.

## Results

Results of the original RF analysis and a log-additive-model logistic regression (LR) analysis are summarized in Table 1. Although most top-ranking SNPs for the two approaches are in the same genes (*HLA-C*, *HLA-B*, *C5/TRAF1*, and *TNF*), SNPs in different genes were also identified. For instance, SNP rs2476601 in *PTPN22 *has the third smallest *p*-value in LR analysis, but does not rank high in the RF analysis. SNPs in *PADI4 *and *VEGFA *have high RF importance measures, but do not have one of the lowest *p*-values. For genes identified by both approaches, different SNPs were often identified as the most relevant. With both approaches, the highest ranking SNPs were in candidate genes rather than in "null genes".

**Table 1:** Top ten ranked SNPs based on logistic regression *p*-values and RF VI (MDA)

SNP (gene)	
**Logistic regression**	**Original RF**

rs2074488 (*HLA-C*)	rs2249742 (*HLA-C*)
rs9461680 (*HLA-C*)	rs2523619 (*HLA-B*)
rs2476601 (*PTPN22*)	rs833069 (*VEGFA*)
rs2523619 (*HLA-B*)	rs2501787 (*PADI4*)
rs3093662 (*TNF*)	rs10116271 (*C5*)
rs3761847 (*TRAF1*)	rs2596503 (*HLA-B*)
rs2156875 (*HLA-B*)	rs12685344 (*C5*)
rs2395471 (*HLA-C*)	rs2395471 (*HLA-C*)
rs13207315 (*HLA-C*)	rs3093662 (*TNF*)
rs7026551 (*C5*)	rs2596501 (*HLA-B*)

Gene-level results based on the original SNP-RF as well as the haplotype RF are summarized in Table 2. Again, haplotypes in candidate genes ranked higher than "null gene" haplotypes, with haplotypes in *HLA-C*, *HLA-B*, and *C5/TRAF1 *having highest VI. Genes that ranked high based on individual SNP importance were also identified when haplotype importance was used.

**Table 2:** Top five ranked genes based on alternative RF approaches

Original RF		Haplotype RF	
	
max VI	mean VI	max VI	mean VI
*HLA-C*	*VEGFA*	*HLA-C*	*C5*
*HLA-B*	*TNF*	*HLA-B*	*TNF*
*VEGFA*	*HLA-A*	*C5*	*HLA-A*
*PADI4*	*HLA-B*	*VEGFA*	*VEGFA*
*C5*	*HLA-C*	*PADI4*	*IL13*

**Figure 1:**
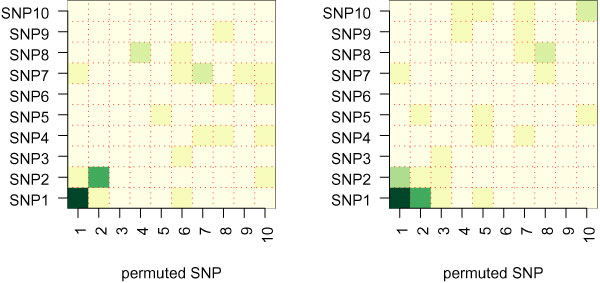
**Application of the gene-permutation method to investigate SNP and interaction importance in simulated data**. Labels along the x-axis identify the permuted SNP. Darker shades of green represent a bigger *DVI*. The first column of each plot shows the changes in variable importance of all SNPs after permuting SNP1 (*DVI*^1^), the second column shows the change in importance after permuting SNP2 (*DVI*^2^), etc. Thus, the diagonal shows  for *k *= *g*, while off diagonal *k *≠ *g*. A, SNP1 and SNP2 have marginal effects but no interaction effect. B, SNPs 1 and 2 interact to influence the probability of disease.

Results of the gene-permutation approach applied to two simulated datasets are shown in Figure 1. As expected, for data simulated under a model with no interaction between the two causal SNPs (SNP1 and SNP2), permutation of SNP1 leads to a large change in importance of SNP1, but not in the importance of SNP2, and vice versa. For data simulated under a model with a strong interaction of SNPs 1 and 2, permuting either one of these SNPs leads to a large difference in variable importance for the other causal SNP. When a similar gene-permutation approach was applied to the RA data, rankings based on differences in VI for SNPs in the permuted gene (, where *k *= *g*) were very similar to the original SNP RF rankings. However, removing the effects of any particular gene via permutation did not have a strong impact on the importance of SNPs in any *other *genes (i.e., , where *k *≠ *g*, were low for all *k *and *g*). Thus, this approach did not detect any important gene-gene interactions in the NARAC data.

## Discussion

We applied variations of RFs to study 17 RA candidate genes and 20 genes not believed to be related to RA. As expected, on average candidate genes ranked higher in terms of RF VI than "null" genes. The RF with SNPs as predictors generally identified the same genes as LR, but often different SNPs in those genes produced the top ranking signals. One potential reason is that for the LR analysis log-additive SNP effects were assumed, while the RF analysis made no assumption about the mode of inheritance of the disease. Also, the RF VI measure that we used, the permutation-based mean decrease in prediction accuracy, is expected to be biased towards more common risk alleles, because SNPs with low minor allele frequencies contribute to fewer cases in the population and thus tend to have lower VI. Another difference between RF and LR that may have contributed to our results is that RF analysis takes into account interactions with other variables. This potentially includes interactions with SNPs in the same gene, which may reflect haplotype effects. Thus RFs, as opposed to single SNP LR analysis, may be better suited to identifying SNPs that impact disease via haplotype effects. This is in line with our finding that genes ranked as most important based on single-SNP RF analysis also ranked highly based on a haplotype RF analysis. Simulations are needed to further investigate these potential causes of differences in results between LR and RF analysis.

It has been suggested that one of the key advantages of RFs is that VI measures capture both main and interaction effects; however, this has not been proven empirically using large genetic datasets. Our gene-permutation strategy did not reveal strong gene-gene interactions. Several reasons may be postulated. First, perhaps the analyzed SNPs do not interact substantially in their effect on RA risk. This is a possibility, given the fact that candidate genes were selected based on previous association studies that focused on single-gene tests. Also, standard RFs may not be optimal for detection of interacting variables in highly dimensional data arising from genetic studies. Further investigation of these possibilities is necessary because the success of future genetic studies depends on using methods that are best suited to the true underlying disease models.

## Conclusion

RFs are a promising approach for genetic data analysis, and extensions beyond simple SNP analyses may enhance their ability to detect predictors of complex diseases. Improved computational strategies are needed to apply these methods on a genome-wide scale, as well as simulation studies comparing the novel RF approaches with traditional statistical methods.

## List of abbreviations used

LR: Logistic regression; NARAC: North American Rheumatoid Arthritis Consortium; RA: Rheumatoid arthritis; RF: Random forest; SNP: Single-nucleotide polymorphism; VI: Variable importance.

## Competing interests

The authors declare that they have no competing interests.

## Authors' contributions

JMB conceived of the study, participated in the design and coordination, and drafted the manuscript. RT ran the statistical analyses and significantly contributed to designing the study and drafting the manuscript. JPS imputed the data using MACH and helped prepare the data for analysis. DNR provided bioinformatics support and selected SNPs for analysis. JL and MdA selected the candidate genes and participated in study design. All authors read, provided feedback, and approved the final manuscript.

## References

[B1] Marchini J Donnelly P Cardon LR Genome-wide strategies for detecting multiple loci that influence complex diseases Nat Genet 2005 37 413 417 10.1038/ng1537 15793588

[B2] Ziegler A DeStefano AL König IR Bardel C Brinza D Bull S Cai Z Glaser B Jiang W Lee KE Li CX Li J Li X Majoram P Meng Y Nicodemus KK Platt A Schwarz DF Shi W Shugart YY Stassen HH Sun YV Won S Wang W Wahba G Zagaar UA Zhao Z Data mining, neural nets, trees--problems 2 and 3 of Genetic Analysis Workshop 15 Genet Epidemiol 2007 31 suppl 1 S51 60 10.1002/gepi.20280 18046765

[B3] Bureau A Dupuis J Falls K Lunetta KL Hayward B Keith TP Van Eerdewegh P Identifying SNPs predictive of phenotype using random forests Genet Epidemiol 2005 28 171 182 10.1002/gepi.20041 15593090

[B4] Bureau A Dupuis J Hayward B Falls K Van Eerdewegh P Mapping complex traits using Random Forests BMC Genet 2003 4 suppl 1 S64 14975132 10.1186/1471-2156-4-S1-S64 PMC1866502

[B5] Lunetta KL Hayward LB Segal J Van Eerdewegh P Screening large-scale association study data: exploiting interactions using random forests BMC Genet 2004 5 32 15588316 10.1186/1471-2156-5-32 PMC545646

[B6] Breiman L Random forests Mach Learn 2001 45 5 32 10.1023/A:1010933404324

[B7] Chen X Liu CT Zhang M Zhang H A forest-based approach to identifying gene and gene gene interactions Proc Natl Acad Sci USA 2007 104 19199 19203 18048322 10.1073/pnas.0709868104 PMC2148267

[B8] Nonyane BAS Foulkes AS Multiple imputation and random forests (MIRF) for unobservable, high-dimensional data Int J Biostat 2007 3 1 18 10.2202/1557-4679.1049 22550652

[B9] Li Y Abecasis GR Mach 1.0: rapid haplotype reconstruction and missing genotype inference [abstract 2290/C] Am J Hum Genet 2006 S79 416

[B10] Liaw A Wiener M Classification and regression by randomForest R News 2002 2 18 22

[B11] Sinnwell J Schaid D Statistical analysis of haplotypes with traits and covariates when linkage phase is ambiguous R package version 1.3.8 2008 http://mayoresearch.mayo.edu/mayo/research/schaid_lab/software.cfm

